# Supplementary material for: Enhanced Energy Density for P-Doped Hierarchically Porous Carbon-Based Symmetric Supercapacitor with High Operation Potential in Aqueous H2SO4 Electrolyte
Source: Nanomaterials (Basel). 2021 Oct 25;11(11):2838. doi: 10.3390/nano11112838 (PMC8624919; doi:10.3390/nano11112838)
Supplement: Supplementary file 1 [file nanomaterials-11-02838-s001.zip › nanomaterials-1388851-supplementary.pdf]

supplementary materials

Enhanced Energy Density for P-Doped Hierarchically Porous Carbon-Based Symmetric Supercapacitor with High Operation Potential in Aqueous H_2SO_4 Electrolyte

Xiaozhong Wu [1], Xinping Yang [1], Wei Feng [2], Xin Wang [1], Zhichao Miao [1], Pengfei Zhou [1], Jinping Zhao [1], Jin Zhou [1] and Shuping Zhuo [1,*]

[1] School of Chemistry and Chemical Engineering, Shandong University of Technology, Zibo 255049, China; xiaozhongwu@yeah.net (X.W.), xinpingyang996@163.com (X.Y.), wang0126xin@163.com (X.W.), miaozhichao@sdut.edu.cn (Z.M.), pengfeizhou1231@126.com (P.Z.), jpzhao@sdut.edu.cn (J.Z.), zhoujin@sdut.edu.cn (J.Z.)
[2] Shandong Qilu Keli Chemical Institute Co., Ltd., Zibo 255086, China; Fengwei007@126.com
* Correspondence: zhuosp_academic@yahoo.com

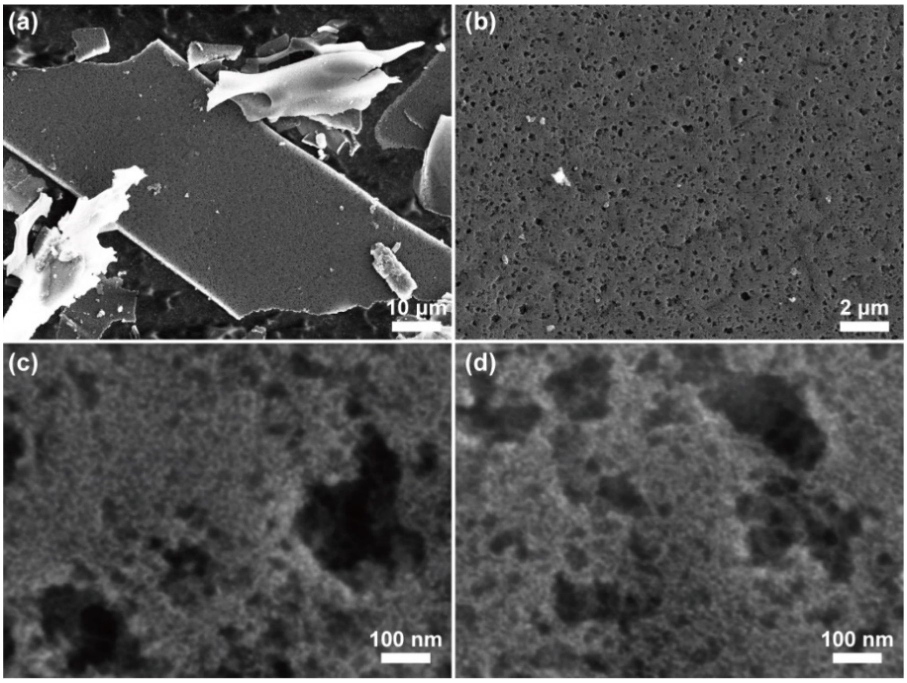

Figure S1. SEM images for HPC6.

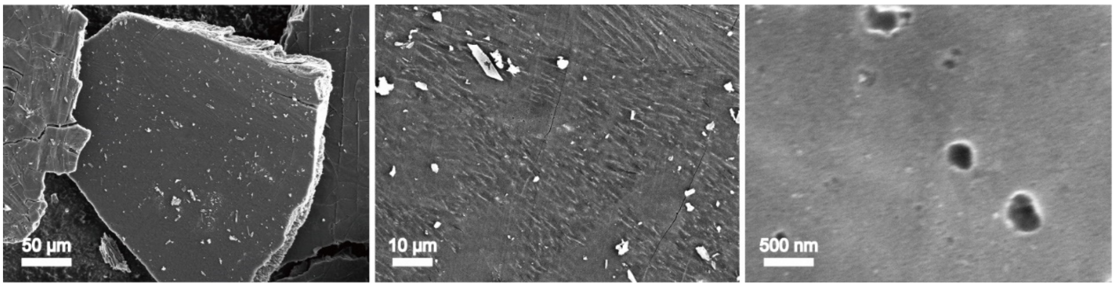

Figure S2. SEM images for C8.

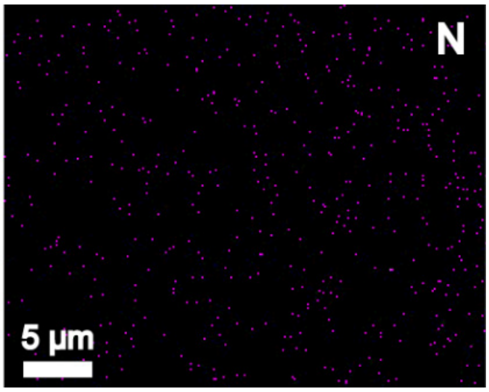

Figure S3. EDS element mapping of nitrogen for HPC8.

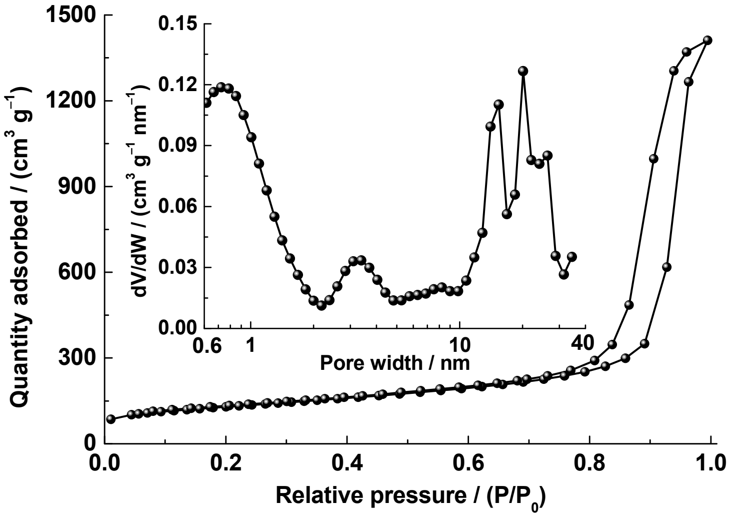

Figure S4. Nitrogen sorption isotherm and QSDFT pore size distribution (inset) for C8.

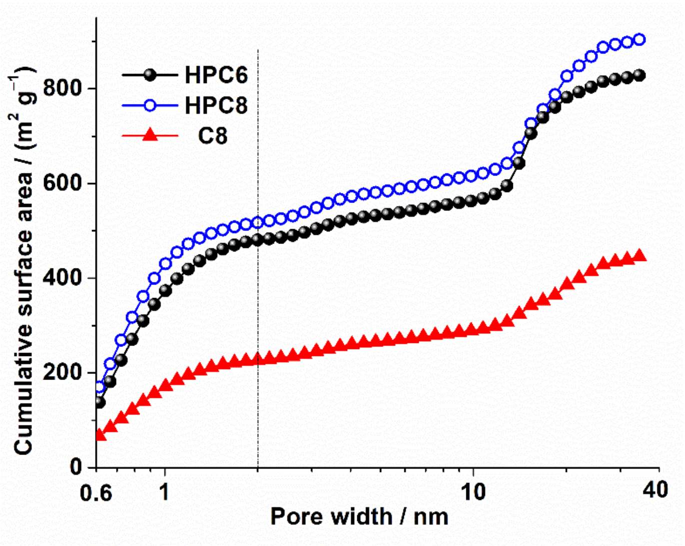

Figure S5. Cumulative surface areas derived from QSDFT method for C8 and HPCs.

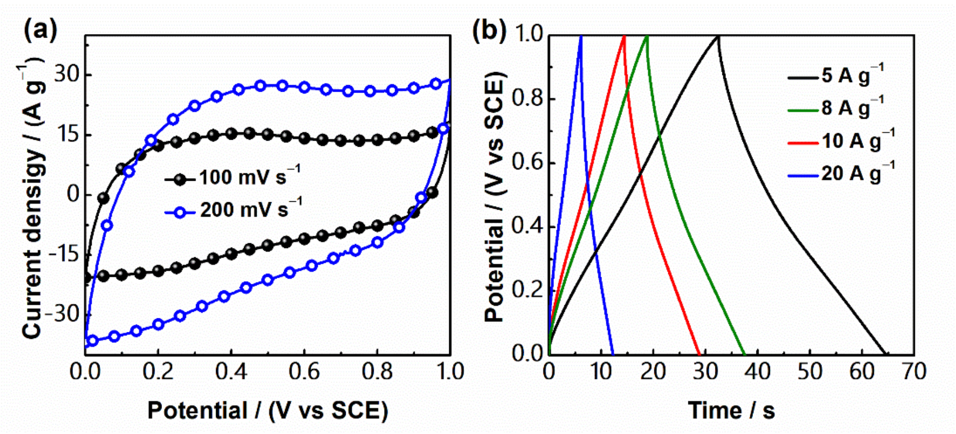

Figure S6. Supercapacitive performance for HPC8 in 1 M H_2SO_4 electrolyte with a three-electrode system: (a) CV curves at different scan rates, (b) GCD curve at different current densities.

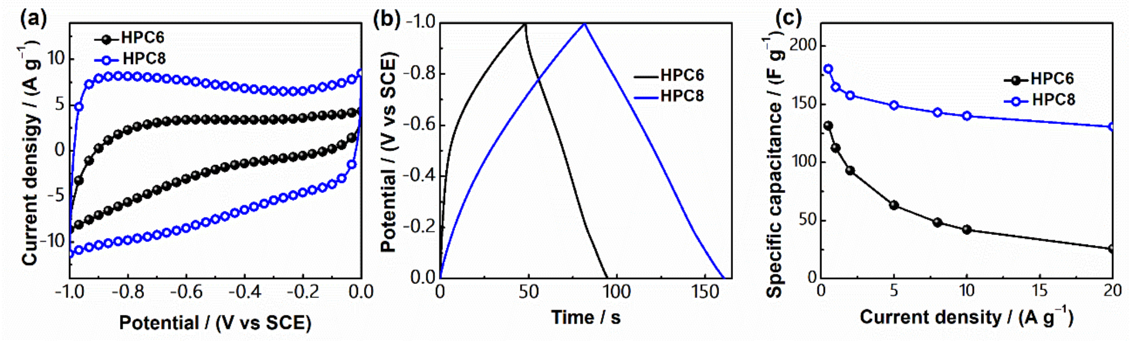

Figure S7. Supercapacitive performance for HPCs in 6 M KOH electrolyte with a three-electrode system: (a) CV curves at a scan rate of 50 mV s^{-1}, (b) GCD curve at a current density of 2 A g^{-1}, (c) specific capacitances at different current densities.

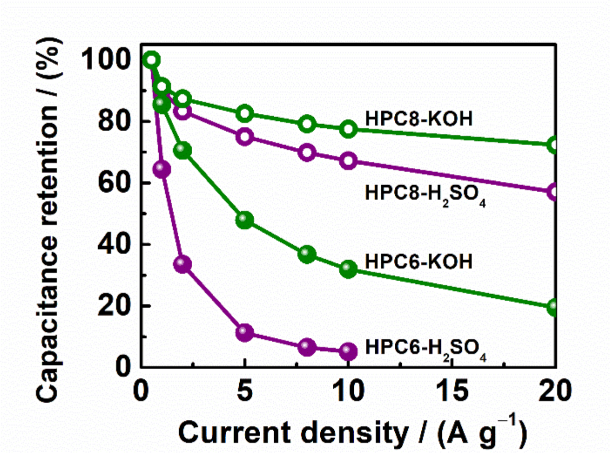

Figure S8. Capacitance retention ratio for HPCs in KOH and H_2SO_4 electrolytes.

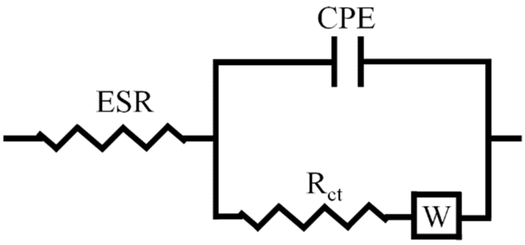

Figure S9. The equivalent circuit for HPC8 in H_2SO_4 electrolyte.

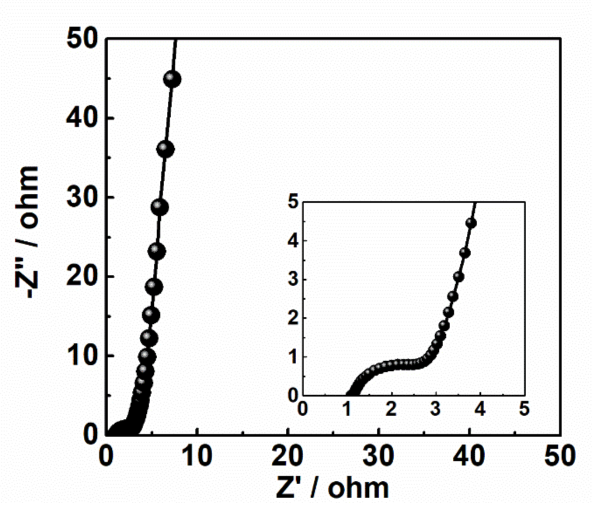

Figure S10. Nyquist plot for HPC8-based symmetric supercapacitor.

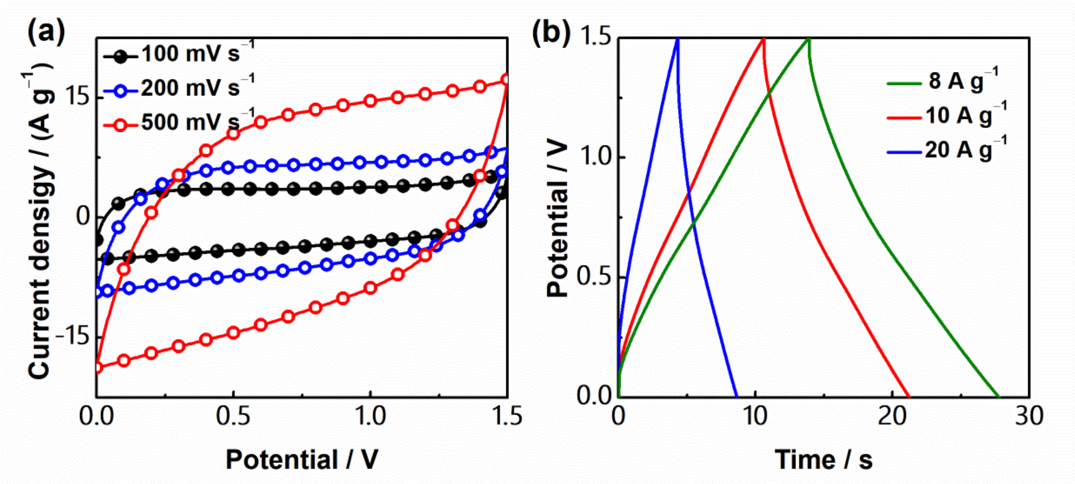

Figure S11. Supercapacitive performance for HPC8-based symmetric supercapacitor: (a) CV curves at different scan rates, (b) GCD curves at different current densities.

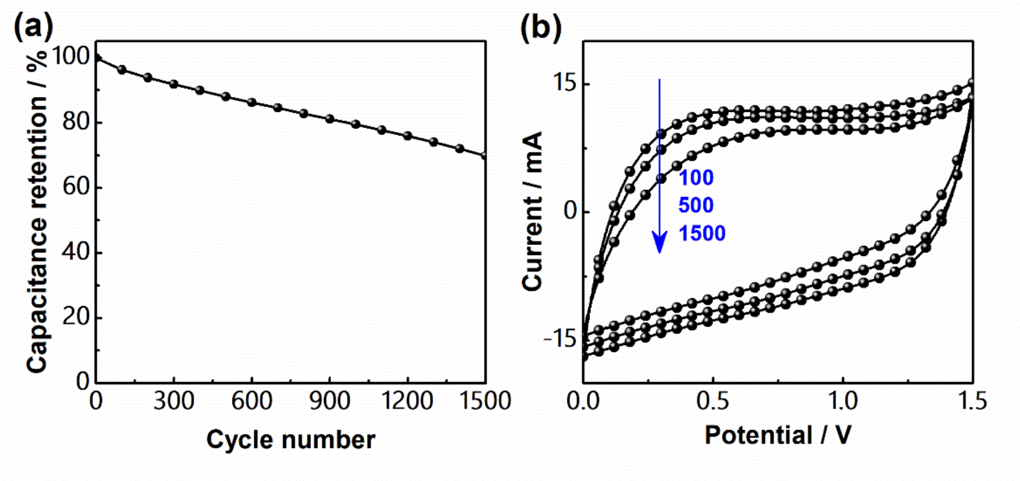

Figure S12. (a) Cyclic stability for HPC8-based symmetric supercapacitor, (b) CV curves (inset) at different cycles.

Table S1. The fitted resistances for HPCs in H_2SO_4 electrolyte.

Sample	ESR	R_{ct}	Z_w
		[Ω]	
**HPC8**	0.51	1.5	1.5
